# Overexpression of the endogenous diguanylate cyclase gene *dgcS* enhances biofilm formation by *Shewanella oneidensis* MR-1 on electrodes

**DOI:** 10.3389/fmicb.2026.1957821

**Published:** 2026-10-14

**Authors:** Nanae Iwanami, Akiho Matsumoto, Ryotaro Yamamoto, Keisuke Tomita, Atsushi Kouzuma, Kazuya Watanabe

**Affiliations:** 1 School of Life Sciences, Tokyo University of Pharmacy and Life Sciences, Hachiōji, Japan; 2 Faculty of Life Sciences, Toyo University, Asaka, Japan

**Keywords:** biofilms, c-di-GMP, cyclic AMP (cAMP), extracellular electron transfer (EET), *Shewanella oneidensis*

## Abstract

In microbial electrochemical technologies, *Shewanella oneidensis* MR-1 efficiently generates electric current via biofilm formation on electrodes. A previous study demonstrated that the deletion of the diguanylate cyclase gene *dgcS* inhibits biofilm formation on electrodes and current generation in MR-1. However, the molecular mechanisms by which *dgcS* expression and subsequent cyclic dimeric guanosine monophosphate (c-di-GMP) signaling affect these processes remain unexplored. Here, we investigated the effects of *dgcS* overexpression on current generation, electrode-associated biofilm formation, and the intracellular concentration of cyclic AMP (cAMP), a second messenger that induces the expression of extracellular electron transfer (EET)-related genes in MR-1, to examine the potential coordinated regulation of intracellular c-di-GMP and cAMP levels. In an electrochemical flow cell (EFC), the *dgcS*-overexpressing (*dgcS*-OE) strain exhibited significantly higher intracellular c-di-GMP concentrations than the vector control (VC) strain, forming more biomass-rich biofilms on the electrode and generating higher current. However, current generation normalized to electrode-associated biomass did not differ significantly between the *dgcS*-OE and VC strains, suggesting that *dgcS* overexpression did not increase EET activity per biomass. Moreover, intracellular cAMP levels were not significantly different between the *dgcS*-OE and VC strains, and the transcription levels of adenylate cyclase (cAMP synthase) genes and EET-related genes in the *dgcS*-OE strain tended to be reduced to 0.3- to 0.6-fold of those in the VC strain. Collectively, these findings suggest that *dgcS* overexpression and the associated elevation of intracellular c-di-GMP levels enhance the electrogenic performance of MR-1 primarily by promoting biofilm development on electrodes, while weakly repressing the transcription of genes related to cAMP signaling.

## Introduction

Electrochemically active bacteria (EAB) couple intracellular metabolic redox reactions to the reduction or oxidation of extracellular solid materials, such as insoluble metal oxides and electrodes (Logan et al., 2019). Most well-studied, Gram-negative EAB perform extracellular electron transfer (EET) via a network of multiheme *c*-type cytochromes (*c*-Cyts) spanning the inner membrane, periplasm, and outer membrane (OM) (Shi et al., 2016). Due to this bioelectrochemical capability, EAB have attracted considerable interest for applications in microbial electrochemical technologies (METs), such as microbial fuel cells (MFCs) (Mahto and Das, 2025), microbial electrosynthesis (Rabaey and Rozendal, 2010), and electro-fermentation (Yamada et al., 2021). In METs, particularly those operated under continuous medium-flow conditions, electrode-associated biofilms (EBFs) formed by EAB serve as efficient catalysts for energy conversion at the cell–electrode interface (Borole et al., 2011). Thus, elucidating the mechanisms governing EBF formation is essential for optimizing MET performance.

*Shewanella oneidensis* MR-1 is a model EAB widely used to investigate the molecular mechanisms of EET and current generation due to its genetic tractability and ease of cultivation (Kouzuma, 2021). In the EET pathway of MR-1, the OM-localized *c*-Cyt complex MtrCAB and the peripheral *c*-Cyt OmcA function as terminal reductases for insoluble extracellular electron acceptors (Fredrickson et al., 2008; Edwards et al., 2020). Consequently, the transcription levels of these EET component genes are an important factor affecting the bioelectrochemical activity of MR-1 (Kasai et al., 2019; Liu et al., 2025). Previous studies have demonstrated that the expression of *omcA* and *mtrCAB* is activated by the cyclic AMP (cAMP) receptor protein (CRP) in cooperation with cAMP (Saffarini et al., 2003; Charania et al., 2009; Kasai et al., 2015). However, the specific environmental stimuli that trigger the expression of these EET-related genes remain unknown.

Current generation in METs depends on the biomass, architecture, and composition of EBFs, as well as the EET activity per cell (Borole et al., 2011). However, the regulatory mechanisms underlying EBF development remain poorly understood, hindering comprehensive insights into current-generation dynamics in METs. To investigate EBFs formed under medium-flow conditions, we previously developed an electrochemical flow cell (EFC) (Kitayama et al., 2017). Using this system, we demonstrated that MR-1 modulates its EBF structure and gene expression profiles, including the upregulation of the diguanylate cyclase (DGC) gene *dgcS* (SO_1646), in response to the presence or absence of medium flow (Kitayama et al., 2017; Koga et al., 2020). DgcS synthesizes cyclic dimeric guanosine monophosphate (c-di-GMP), a second messenger that mediates the transition from a planktonic to a biofilm lifestyle in proteobacteria (Matsumoto et al., 2021). Deletion of *dgcS* in MR-1 reduces intracellular c-di-GMP levels, impairing EBF formation and current generation, suggesting that DgcS is a primary determinant of c-di-GMP levels under current-generating conditions with medium flow (Matsumoto et al., 2021). Furthermore, heterologous expression of a DGC gene in MR-1 has been reported to increase the expression of *omcA* and *mtrC*, implying a regulatory link between c-di-GMP signaling and EET activity (Ng et al., 2020). However, that analysis was conducted using aerobically grown cells, and how c-di-GMP signaling via endogenous DGCs affects EET-related gene expression in MR-1 cells grown under anaerobic, current-generating conditions remains unelucidated.

In this study, we constructed a *dgcS*-overexpressing strain to investigate how elevated c-di-GMP levels originating from the endogenous DGC modulate EBF formation and current generation in MR-1. Furthermore, we quantified intracellular c-di-GMP and cAMP levels and determined the expression levels of EET-related genes under multiple anaerobic cultivation conditions to explore the potential cross-regulation between EBF development and EET activity.

## Materials and methods

### Bacterial strains and culture conditions

Strains and plasmids are listed in Supplementary Table 1. *S. oneidensis* strains were cultured at 30 °C in Lysogeny Broth (LB) medium or lactate minimal medium (LMM) containing 15 mM DL-lactate as the sole carbon and energy source (Kitayama et al., 2017). For aerobic cultures, a test tube containing LB medium or LMM was inoculated with an *S. oneidensis* strain at an initial optical density at 600 nm (OD_600_) of 0.05 and incubated with reciprocal shaking at 180 rpm. For anaerobic cultures, a screw-top test tube containing LMM supplemented with 50 mM fumarate as the electron acceptor was inoculated with an *S. oneidensis* strain at an initial OD_600_ of 0.01. The test tube was then sealed with a butyl rubber septum, purged with high-purity nitrogen (99.99%) for 20 min, and incubated without shaking. The OD_600_ was monitored using an OD-monitor C&T (Taitec, Saitama, Japan). *Escherichia coli* strains were cultivated in LB medium at 37 °C. The *E. coli* donor strain WM6026 was grown in medium supplemented with 100 μg/mL 2,6-diaminopimelic acid (DAP). When necessary, 50 μg/mL kanamycin (Km) or 15 μg/mL gentamicin (Gm) was added to the culture medium. Solid media were prepared by adding 1.6% (w/v) Bacto agar (Difco, Franklin Lakes, NJ, USA).

### Construction of transformants

The *dgcS*-expressing plasmid pBBR-*dgcS* (Matsumoto et al., 2021) and the control vector pBBR1MCS-2 (Kovach et al., 1995) were introduced into MR-1 by filter mating with *E. coli* WM6026, as described previously (Matsumoto et al., 2021). The resulting strains harboring pBBR-*dgcS* and pBBR1MCS-2 were designated *dgcS*-OE and VC, respectively. For biofilm observation in EFCs, a plasmid expressing an anaerobic fluorescent protein (AFP), pME*lacZ*-AFP, was introduced into the *dgcS*-OE strain by electroporation, as described previously (Kitayama et al., 2017). The resulting transformant was designated *dgcS*-OE_*AFP*_. MR-1 harboring pBBR1MCS-2 and pME*lacZ*-AFP, constructed in a previous study (Matsumoto et al., 2021), is referred to as VC_*AFP*_ in this study.

### EFC operation and biofilm observation

The setup and operation of EFCs were performed according to methods described previously (Kitayama et al., 2017). Briefly, a three-electrode EFC comprising a graphite plate working electrode (68 × 140 mm; IG-70; Toyo Tanso, Osaka, Japan), an Ag/AgCl reference electrode (HX-R5; Hokuto Denko, Tokyo, Japan), and a titanium mesh counter electrode (25 × 25 mm, 100 mesh, eight pieces; Nilaco, Tokyo, Japan) was filled with LMM containing Km and Gm, inoculated with either the *dgcS*-OE_*AFP*_ or VC_*AFP*_ strain at an initial OD_600_ of 0.003, and incubated for 1 h to allow initial cell attachment prior to the initiation of medium flow. The electrodes were connected to a potentiostat (VMP3; Bio-Logic Science Instruments, Seyssinet-Pariset, France), and the current was monitored at a working electrode potential of +0.4 V versus the standard hydrogen electrode (SHE). Current density (A/cm^2^) was calculated based on the projected area of the working electrode exposed to the EFC interior (50 cm^2^). For EFC operation under medium-flow conditions, LMM containing Km and Gm was continuously supplied from a reservoir tank to the EFC using a peristaltic pump at a constant linear velocity of 5.5 mm min^–1^ (corresponding to a flow rate of 1.38 mL min^–1^). The medium in the reservoir tank was constantly purged with high-purity nitrogen gas to maintain anaerobic conditions. During operation, the EFC was placed in an incubator at 30 °C. Statistical differences in maximum current densities between strains were analyzed using Welch’s *t*-test (*n* = 3 biological replicates). Confocal laser scanning microscopy (CLSM) was performed using an FV1200 (BX61WI configuration) microscope system (Olympus, Tokyo, Japan) according to a method described previously (Kitayama et al., 2017). High-resolution images were obtained using FV-OSR technology (Olympus) and processed with imaging software (Fluoview v4.2; Olympus). Biofilms expressing AFP were visualized at an excitation wavelength of 473 nm using a DM473 dichroic mirror and BA490-525 emission filters. Reproducibility was validated in three independent EFC runs, and representative images are shown in the Results.

### Batch EC operation

A single-chamber three-electrode EC with a capacity of 18 mL (Hirose et al., 2018) was used to monitor the electric current generated by *S. oneidensis* strains under batch conditions. The EC was equipped with a graphite felt working electrode (10 × 10 mm), an Ag/AgCl reference electrode (HX-R5; Hokuto Denko), and a platinum wire counter electrode (5 cm, φ0.3 mm, Nilaco). The EC was filled with 15 mL of LMM, inoculated with bacterial cells at an initial OD_600_ of 0.01, and purged with high-purity nitrogen (99.99%). The electrodes were connected to a potentiostat (VMP3; Bio-Logic Science Instruments), and the working electrode was poised at +0.4 V versus SHE. The system was operated in an incubator at 30 °C. Current density (A/cm^2^) was calculated based on the single-sided projected area of the working electrode (1.0 cm^2^). Statistical differences in maximum current densities between strains were analyzed using Welch’s *t*-test (*n* = 3 biological replicates).

### Quantification of polysaccharides and proteins in EFC biofilms

Electrochemical flow cell biofilms were recovered using a silicone scraper from a 30-cm^2^ central area of the working electrode (located between the medium inlet and outlet, an area exposed to effective medium flow) after 168 h of operation. Polysaccharides and proteins were extracted from separate EFC runs and quantified using the phenol-sulfuric acid method (DuBois et al., 1956) and a Micro BCA Protein Assay Kit (Thermo Fisher Scientific, Waltham, MA, USA), respectively, as described previously (Kitayama et al., 2017). Statistical differences in protein and polysaccharide content between strains were analyzed using Welch’s *t*-test (*n* = 3 biological replicates). The polysaccharide/protein ratio was calculated using the respective group means, and statistical differences between strains were evaluated using the Delta method.

### Quantification of intracellular c-di-GMP and cAMP levels

Cell lysates for c-di-GMP and cAMP quantification were prepared from cells harvested from 2-mL cultures of *S. oneidensis* anaerobically grown under fumarate-reducing conditions for 8 h (corresponding to the early stationary phase), EFC biofilm cells harvested from the central area of the working electrode (30 cm^2^) after 72 h of operation, or batch EC electrode-associated cells collected from the working electrode (1.0 cm^2^) after 48 h of operation. Cells from the fumarate-reducing cultures were harvested by centrifugation at 13,000 × *g* for 1 min at 4 °C. EFC biofilms were scraped off using a silicone scraper and collected into microcentrifuge tubes. The working electrodes of the batch ECs were removed from the reactors and placed directly into microcentrifuge tubes. These samples were prepared on ice and stored at −80 °C prior to c-di-GMP and cAMP quantification. For c-di-GMP quantification, the samples were suspended in B-PER Reagent (Thermo Fisher Scientific), using 200 μL for fumarate-reducing culture cells and EFC biofilms, or 1 mL for batch EC electrode-associated cells. The c-di-GMP concentration in the resulting cell lysates was determined using a Cyclic di-GMP ELISA Kit (Cayman Chemical, Ann Arbor, MI, USA) according to the manufacturer’s instructions. Intracellular cAMP levels were quantified as previously described (Kasai et al., 2018). Briefly, the collected samples were suspended in a lysis buffer containing 100 μM Ro20-1724, 500 μM 3-isobutyl-1-methylxanthine, 50 mM HEPES-NaOH (pH 7.4), and 5% Triton X-100, using the same respective volumes as described above. The cAMP concentration in the resulting lysates was determined using a Cyclic AMP EIA Kit (Cayman Chemical) according to the manufacturer’s instructions. The measured c-di-GMP and cAMP levels were normalized to the total protein content of each sample, determined using a Micro BCA Protein Assay Kit (Thermo Fisher Scientific), and statistical differences between strains were analyzed using Welch’s *t*-test (*n* = 3 biological replicates).

### RNA extraction

Total RNA was extracted using TRIzol Reagent (Thermo Fisher Scientific) from cells harvested from *S. oneidensis* cultures anaerobically grown under fumarate-reducing conditions for 8 h, EFC biofilm cells harvested from the working electrode after 72 h of operation, or batch EC electrode-associated cells collected from the working electrode after 48 h of operation. The extracted RNA was purified using an RNeasy Mini Kit and an RNase-free DNase Set (Qiagen, Valencia, CA, USA), and its quality was evaluated using an Agilent 2100 Bioanalyzer with RNA 6000 Pico reagents and RNA Pico Chips (Agilent Technologies, Santa Clara, CA, USA) according to the manufacturer’s instructions.

### RT-qPCR

Reverse transcription quantitative PCR (RT-qPCR) was performed according to a method described previously (Koga et al., 2020). Briefly, total RNA was reverse-transcribed using SuperScript III Reverse Transcriptase (Thermo Fisher Scientific) and Random Primers (Thermo Fisher Scientific) according to the manufacturer’s instructions. Quantitative PCR was performed using a StepOnePlus or QuantStudio 1 Real-Time PCR system (Thermo Fisher Scientific). The PCR reaction mixture (20 μL) contained 2 μL cDNA solution, 10 μL Power SYBR Green PCR Master Mix (Thermo Fisher Scientific), and 0.5 μM of each primer listed in Supplementary Table 2. The expression level of each gene was quantified using the standard curve method, where standard curves were generated using 10-fold serial dilutions of purified target gene fragments. Specificity of the amplification was verified by dissociation-curve analysis and gel electrophoresis of the PCR products. Fluorescence signals in no-template controls and no-reverse-transcription controls were negligible. The expression levels of the target genes were normalized to that of the 16S rRNA gene, and statistical differences in relative expression levels between strains were analyzed using Welch’s *t*-test (*n* = 3–4 biological replicates).

## Results

### Construction and characterization of the *dgcS*-OE strain

To construct a *dgcS*-overexpressing strain, the plasmid pBBR-*dgcS* (Matsumoto et al., 2021), which expresses *dgcS* under the control of the constitutive *lac* promoter in *Shewanella*, was introduced into *S. oneidensis* MR-1. The resulting strain (designated as *dgcS*-OE) was cultivated in lactate minimal medium (LMM) under aerobic conditions and anaerobic conditions with fumarate as the electron acceptor. Under both conditions, the *dgcS*-OE strain exhibited growth comparable to that of the vector control strain (VC) harboring the empty vector pBBR1MCS-2 (Supplementary Figure 1), suggesting that *dgcS* overexpression does not significantly affect cell proliferation in liquid culture.

Evaluation of *dgcS* expression and c-di-GMP concentrations in cells grown in LMM under fumarate-reducing conditions confirmed that both *dgcS* transcription and c-di-GMP levels were significantly higher in the *dgcS*-OE strain than in the VC strain (Figures 1A, B). To determine whether this elevated intracellular c-di-GMP level alters the concentration of cAMP, a second messenger that triggers the expression of extracellular electron transfer (EET)-related genes (Saffarini et al., 2003), we also compared the intracellular cAMP concentrations in the *dgcS*-OE and VC strains. A *cyaC*-overexpressing (*cyaC*-OE) strain (Kasai et al., 2019), which overexpresses the primary adenylate cyclase in MR-1 (Charania et al., 2009), was utilized as a positive control. Although the *cyaC*-OE strain exhibited a substantial elevation in cAMP levels as previously reported (Kasai et al., 2019), the intracellular cAMP concentration in the *dgcS*-OE strain was comparable to that of the VC strain (Figure 1C). Additionally, the expression levels of the CRP-regulated OM *c*-Cyt genes *omcA* and *mtrC* were not significantly different between *dgcS*-OE and VC cells grown under the fumarate-reducing conditions (Supplementary Figure 2). Taken together, these results indicate that *dgcS* overexpression elevates the intracellular c-di-GMP concentration without altering cAMP levels or upregulating CRP-regulated genes under the cultivation conditions examined (i.e., in liquid LMM under fumarate-reducing conditions).

**FIGURE 1:**
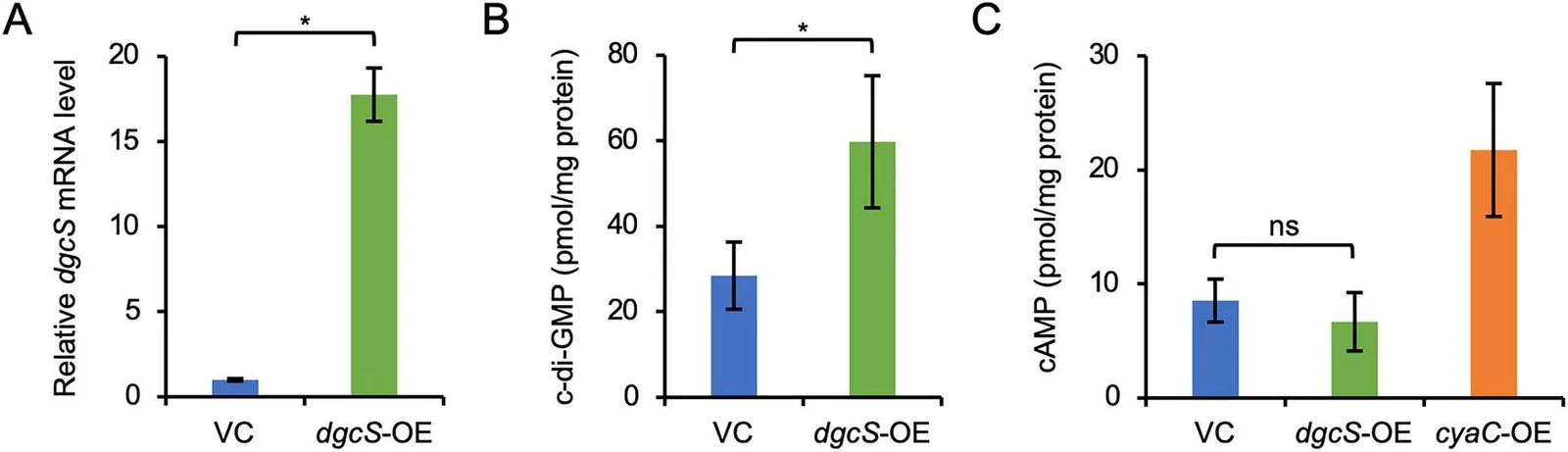
Effects of *dgcS* expression on intracellular cyclic dimeric guanosine monophosphate (c-di-GMP) and cyclic AMP (cAMP) levels in *Shewanella oneidensis* MR-1 grown under fumarate-reducing conditions. **(A)** Comparison of *dgcS* expression levels between the *dgcS*-overexpressing (*dgcS*-OE) and vector control (VC) strains by reverse transcription quantitative PCR (RT-qPCR) analysis. **(B)** Intracellular c-di-GMP levels in the *dgcS*-OE and VC strains. **(C)** Intracellular cAMP levels in the *dgcS*-OE and VC strains. In all panels, bars and error bars represent the means and standard deviations, respectively, calculated from three independent experiments. Asterisks indicate statistically significant differences (*p* < 0.05; Welch’s *t-*test). ns, not significant.

### Biofilm formation and current generation by the *dgcS*-OE strain

To evaluate the impact of *dgcS* expression on EBF formation and current generation, EFCs were inoculated with the *dgcS*-OE_*AFP*_ and VC_*AFP*_ strains, both harboring pME*lacZ*-AFP (Kitayama et al., 2017), a plasmid expressing AFP from the *lac* promoter, and operated under continuous medium-flow conditions. The working electrode potential was maintained at +0.4 V (vs. SHE). The *dgcS*-OE_*AFP*_ strain exhibited a steeper initial current increase than the VC_*AFP*_ strain (Figure 2A), and its maximum current density (3.37 μA/cm^2^) was approximately 2.4-fold higher than that of the VC_*AFP*_ strain (1.40 μA/cm^2^) (Figure 2B). Confocal laser scanning microscopy (CLSM) analysis of the EBFs after 168 h of EFC operation, when current densities had stabilized at their maximum values in both strains, revealed that the cell density of the *dgcS*-OE_*AFP*_ strain was substantially higher than that of the VC_*AFP*_ strain near the working electrode surface (Figures 2C,D; note that excitation laser attenuation limits strict fluorescence quantitation, particularly at the electrode interface). Consistent with these observations, quantification of the total protein content in the EBF cells harvested at 168 h demonstrated that the *dgcS*-OE_*AFP*_ biofilm contained 2.9-fold more biomass than the VC_*AFP*_ biofilm (Figure 2E). These results indicate that the *dgcS*-OE_*AFP*_ strain develops a biofilm with higher cell density on the working electrode, thereby facilitating EET from the cells to the electrode. However, when the maximum current densities (nearly equivalent to those at 168 h; Figure 2A) were normalized to the biofilm protein content, the normalized values showed no significant difference between the *dgcS*-OE_*AFP*_ and VC_*AFP*_ strains (Figure 2F), indicating that the EET activity per biomass was unaffected by *dgcS* overexpression.

**FIGURE 2:**
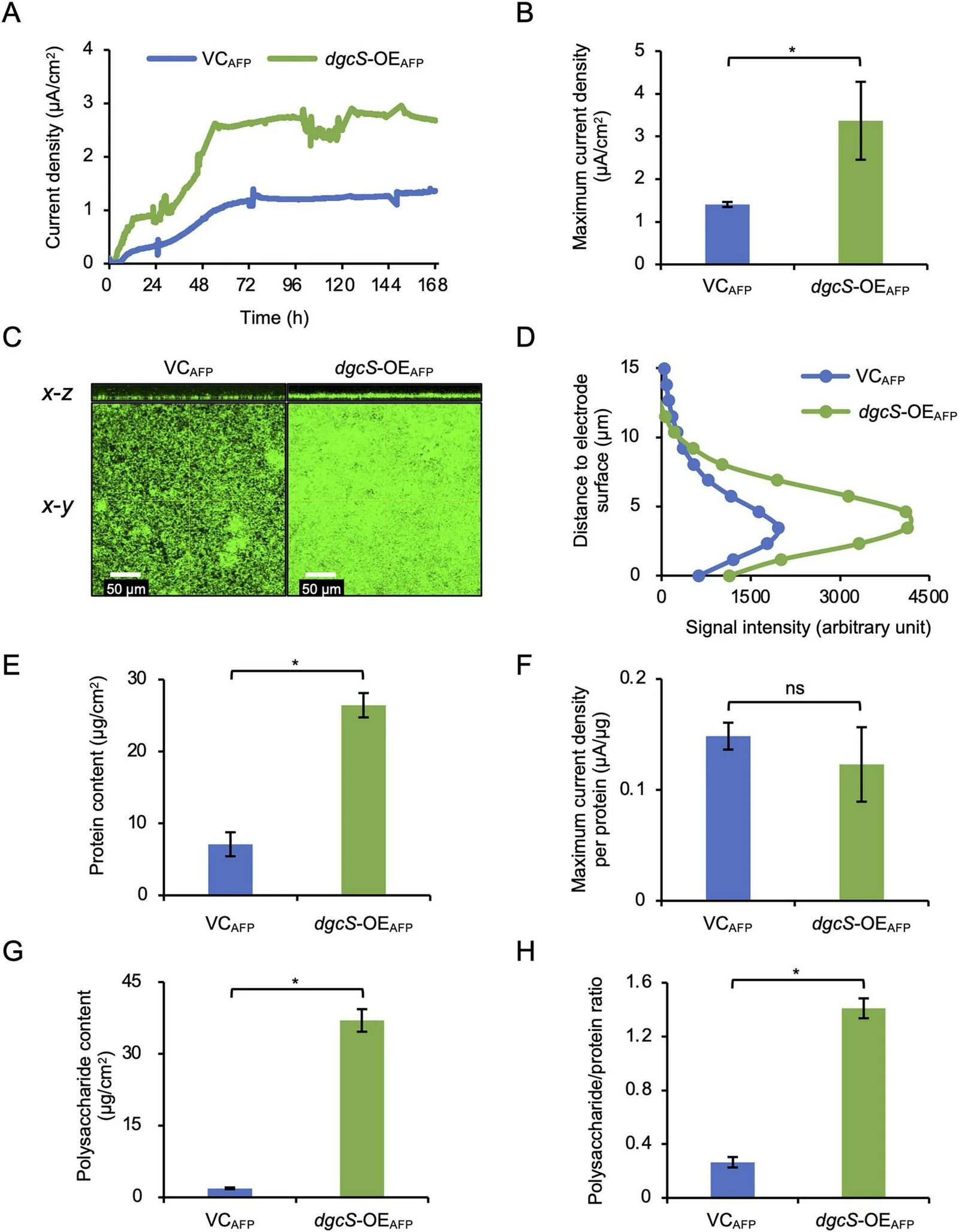
Current generation and electrode-associated biofilm (EBF) formation by the *dgcS*-overexpressing (*dgcS*-OE) strain in electrochemical flow cells (EFCs). **(A)** Typical time courses of current generation by the *dgcS*-OE_*AFP*_ and VC_*AFP*_ strains. **(B)** Comparison of maximum current densities generated by the *dgcS*-OE_*AFP*_ and VC_*AFP*_ strains. **(C)** Representative vertical (*x*-*z*) and horizontal (*x*-*y*) confocal laser scanning microscopy (CLSM) images of the *dgcS*-OE_*AFP*_ and VC_*AFP*_ EBFs developed in EFCs operated for 168 h. The horizontal images were obtained at a height of 3 μm from the working electrode surfaces. **(D)** Vertical distribution of anaerobic fluorescent protein (AFP) signal intensities in CLSM images obtained from EFCs operated for 168 h. **(E–H)** Protein content **(E)**, maximum current density normalized to protein content **(F)**, polysaccharide content **(G)**, and ratios of polysaccharide content to protein content **(H)** in electrode-associated *dgcS*-OE_*AFP*_ and VC_*AFP*_ biofilms. Cells were grown in EFCs for 168 h. In panels **(B,E–G)**, bars and error bars represent the means and standard deviations, respectively (*n* = 3 biological replicates). Asterisks indicate statistically significant differences (*p* < 0.05; Welch’s *t-*test); ns, not significant. In panel **(H)**, the polysaccharide/protein ratio was calculated as the ratio of the respective group means. Error bars represent the standard error of the ratio, estimated using the Delta method. Asterisks indicate statistical significance (*p* < 0.001; two-tailed *Z*-test on log-transformed ratios; *n* = 3 biological replicates). The numerical data for the VC_*AFP*_ strain presented in this figure were previously reported in Matsumoto et al. (2021) and are included here for comparison with the *dgcS*-OE_*AFP*_ strain.

Quantification of polysaccharide content within the EBFs revealed that the *dgcS*-OE_*AFP*_ biofilm contained approximately 20-fold more polysaccharides than the VC_*AFP*_ biofilm (Figure 2G). Even when normalized to the protein content, the polysaccharide-to-protein ratio was significantly higher in the *dgcS*-OE_*AFP*_ strain than in the VC_*AFP*_ strain (Figure 2H). Furthermore, analysis of intracellular c-di-GMP and cAMP levels in cells harvested from the EBFs confirmed that *dgcS*-OE_*AFP*_ cells contained approximately 2-fold more c-di-GMP than VC_*AFP*_ cells, while the cAMP levels in the *dgcS*-OE_*AFP*_ cells did not differ significantly from those in the VC_*AFP*_ cells (Supplementary Figures 3A, B). Collectively, these findings suggest that *dgcS* overexpression promotes polysaccharide synthesis via enhanced intracellular c-di-GMP signaling, thereby facilitating bacterial cell association with the electrode surface.

### Gene expression patterns in *dgcS*-OE biofilm cells

To investigate the impact of *dgcS* expression on the gene expression of MR-1 grown within EBFs, *dgcS*-OE_*AFP*_, and VC_*AFP*_ cells were harvested from the EFC working electrode surface, and their gene expression patterns were analyzed by RT-qPCR for *dgcS* and nine other selected genes (*flgL*, *flrA*, *flrC*, *omcA*, *mtrC*, *cyaA*, *cyaB*, *cyaC*, and *crp*) (Table 1). Among these, *flgL* is involved in flagellar synthesis, and *flrA* and *flrC* are involved in its regulation (Shi et al., 2014; Gao et al., 2018). These genes were selected because flagellar synthesis and motility are known to be repressed by c-di-GMP signaling (Beyhan et al., 2006; Ng et al., 2020). The remaining target genes are CRP-regulated, EET-related genes (*omcA* and *mtrC*), adenylate cyclase (cAMP synthase) genes (*cyaA*, *cyaB*, and *cyaC*), and the *crp* gene itself. The results confirmed a significant upregulation of *dgcS* in EFC-grown *dgcS*-OE_*AFP*_ biofilm cells (Table 1). In contrast, the other target genes tended to be downregulated in the *dgcS*-OE_*AFP*_ strain, although the expression changes of *flrA*, *mtrC*, and *cyaB* were not statistically significant (*p* > 0.05). These results suggest that *dgcS* overexpression leads to the downregulation of genes related to flagellar biosynthesis and cAMP signaling under EFC biofilm conditions, while the repression of the cAMP/CRP-related genes is relatively mild (0.3- to 0.6-fold relative to the control).

**TABLE 1:** Relative expression levels of flagellar biosynthesis and cyclic AMP (cAMP)/cAMP receptor protein (CRP)-related genes in *dgcS*-OE_*AFP*_ and VC_*AFP*_ biofilm cells grown in electrochemical flow cells (EFCs).

Gene	Locus tag	Annotation	Relative expression level^1^		Significance^2^
			*dgcS*-OE_*AFP*_	VC_*AFP*_	
*dgcS*	SO_1646	Diguanylate cyclase DgcS	6.01 ± 2.72	1.00 ± 0.31	*
*flgL*	SO_3239	Flagellar hook-associated protein FlgL	0.12 ± 0.02	1.00 ± 0.17	*
*flrA*	SO_3232	σ^54^-dependent transcriptional regulator for flagellar biosynthesis FlrA	0.49 ± 0.12	1.00 ± 0.56	ns
*flrC*	SO_3230	Two component signal transduction system controlling flagella biosynthesis σ^54^-dependent response regulator FlrC	0.22 ± 0.03	1.00 ± 0.02	**
*omcA*	SO_1779	Extracellular iron oxide respiratory system surface decaheme cytochrome *c* component OmcA	0.49 ± 0.20	1.00 ± 0.24	*
*mtrC*	SO_1778	Extracellular iron oxide respiratory system surface decaheme cytochrome *c* component MtrC	0.59 ± 0.11	1.00 ± 0.17	ns
*cyaA*	SO_4312	Adenylate cyclase CyaA	0.32 ± 0.08	1.00 ± 0.11	**
*cyaB*	SO_3778	Adenylate cyclase CyaB	0.50 ± 0.04	1.00 ± 0.29	ns
*cyaC*	SO_1329	Adenylate cyclase CyaC	0.31 ± 0.14	1.00 ± 0.33	*
*crp*	SO_0624	cAMP-responsive regulator CRP	0.58 ± 0.11	1.00 ± 0.14	*

^1^Relative expression (fold change) was calculated as the ratio of gene expression levels in the *dgcS*-OE_*AFP*_ strain to those in the VC_*AFP*_ strain, as determined by RT-qPCR. Values are presented as the mean ± standard deviation (*n* = 3–4 biological replicates).

^2^Statistical significance was determined using Welch’s *t*-test:

**p < 0.01;

*p < 0.05; ns, not significant (*p* ≥ 0.05).

### Current generation by the *dgcS*-OE strain in batch ECs

To investigate whether the current increase associated with elevated *dgcS* expression is specific to EFC conditions, we evaluated current generation by the *dgcS*-OE and VC strains using a batch EC. Both strains exhibited their maximum current densities at approximately 40–48 h, and the maximum current density of the *dgcS*-OE strain was 1.3-fold higher than that of the VC strain (Figures 3A, B). However, the *dgcS*-OE strain exhibited a slower rate of initial current increase than the VC strain (Figure 3A). This behavior is opposite to the trend observed under medium-flow conditions in the EFC (Figure 2A), suggesting that the impact of *dgcS* overexpression on current generation profiles is influenced by environmental conditions that differ between EFCs and batch ECs.

**FIGURE 3:**
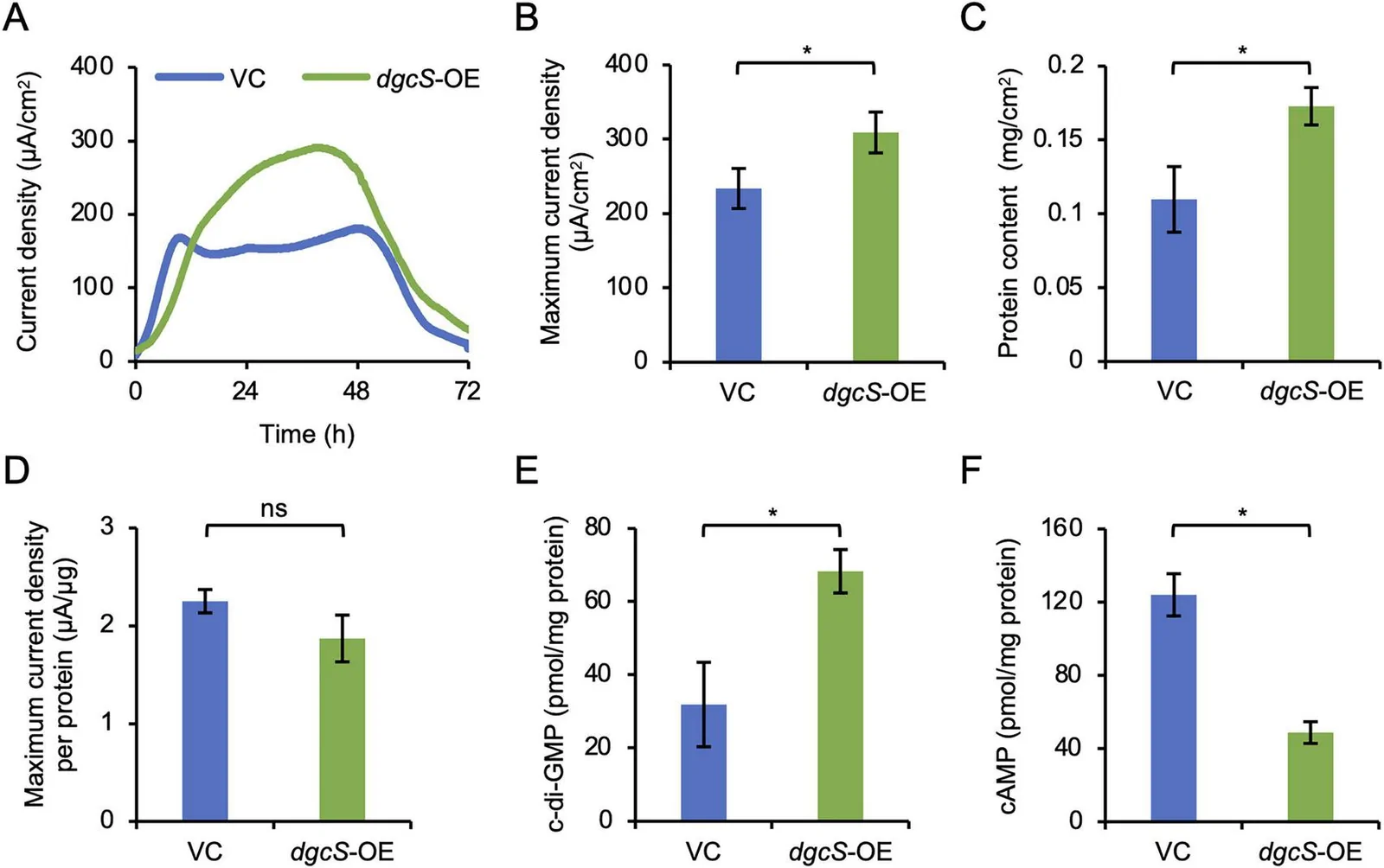
Characterization of the *dgcS*-overexpressing (*dgcS*-OE) strain in batch ECs. **(A)** Typical time courses of current generation by the *dgcS*-OE and vector control (VC) strains. **(B)** Comparison of maximum current densities generated by the *dgcS*-OE and VC strains. **(C–F)** Protein content **(C)**, maximum current density normalized to protein content **(D)**, cyclic dimeric guanosine monophosphate (c-di-GMP) level **(E)**, and cyclic AMP (cAMP) level **(F)** in electrode-associated *dgcS*-OE and VC cells. Bacterial cells were grown in batch ECs for 48 h. In all bar graphs, bars and error bars represent the means and standard deviations, respectively, calculated from three independent experiments. Asterisks indicate statistically significant differences (*p* < 0.05; Welch’s *t*-test). ns, not significant.

Quantification of the total protein content of cells harvested from the working electrode 48 h after the start of batch EC operation (immediately after reaching maximum current density) revealed that the electrode-associated biomass of the *dgcS*-OE strain was 1.6-fold higher than that of the VC strain (Figure 3C). This result confirms that *dgcS* expression promotes bacterial cell association with the electrode even in batch ECs. However, when the maximum current density was normalized to the protein content of the electrode-attached cells, the normalized values were not significantly different between the *dgcS*-OE and VC strains (Figure 3D), consistent with the aforementioned EFC results (Figure 2F). Furthermore, quantification of intracellular c-di-GMP and cAMP levels in the electrode-associated cells harvested at 48 h revealed that while the c-di-GMP concentration in the *dgcS*-OE strain was 2.1-fold higher than in the VC strain (Figure 3E), the cAMP concentration was approximately 0.4-fold lower (Figure 3F). The RT-qPCR analysis confirmed significant *dgcS* overexpression in electrode-associated *dgcS*-OE cells grown in batch ECs, whereas the expression levels of *flrA* and *mtrC* in *dgcS*-OE cells were 0.16- and 0.49-fold of those in VC cells, respectively (Figure 4). Collectively, these results suggest that even in batch ECs, *dgcS* expression contributes to enhanced biofilm formation on electrodes, while its influence on cAMP signaling and associated gene expression is relatively limited.

**FIGURE 4:**
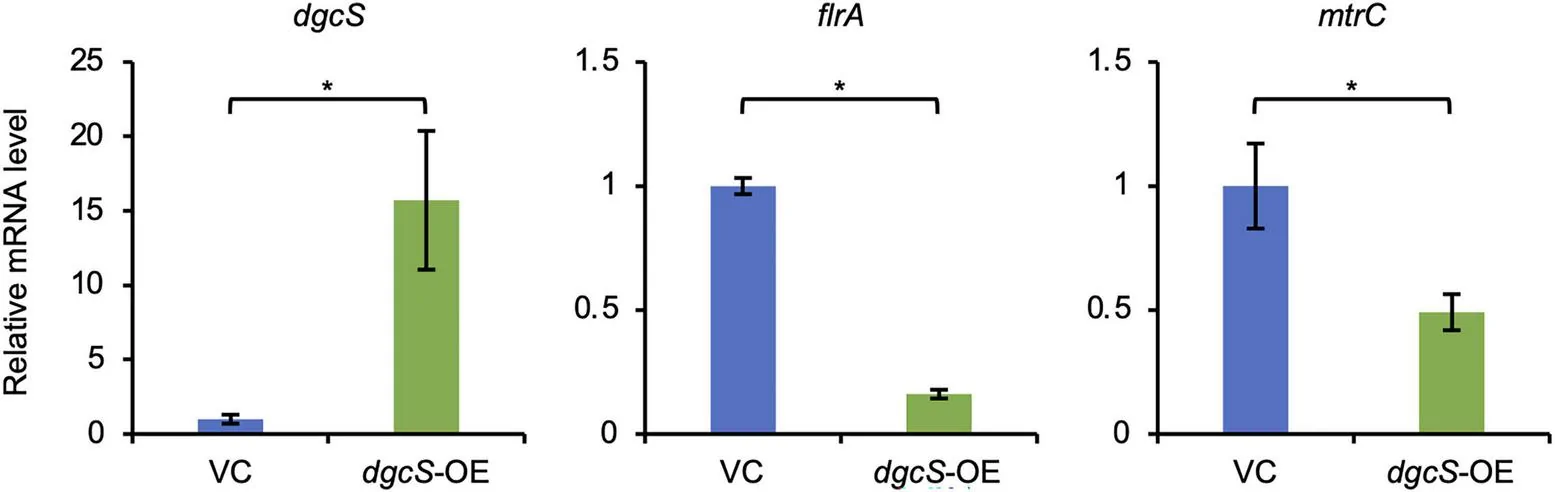
Reverse transcription quantitative PCR (RT-qPCR) analysis of *dgcS*, *flrA*, and *mtrC* in electrode-associated *dgcS*-overexpressing (*dgcS*-OE) and vector control (VC) cells grown in batch ECs operated for 48 h. Bars and error bars represent the means and standard deviations, respectively, calculated from three independent experiments. Asterisks indicate statistically significant differences (*p* < 0.05; Welch’s *t-*test).

## Discussion

In *Pseudomonas aeruginosa* and *Vibrio cholerae*, elevated intracellular c-di-GMP levels repress flagellar biosynthesis, thereby promoting the transition from a planktonic to a biofilm lifestyle (Beyhan et al., 2006; Krasteva et al., 2010; Matsuyama et al., 2016). Our analysis reveals that MR-1 also exhibits a similar response to increased c-di-GMP levels, as evidenced by the downregulation of flagella-related genes (Table 1). Flagella generally play a crucial role during the early stages of biofilm formation by facilitating bacterial migration toward and initial contact with solid surfaces (O’Toole and Kolter, 1998; Hershey, 2021). Notably, in batch ECs, the *dgcS*-OE strain exhibited a slower initial increase in current generation than the VC strain, in contrast to the profiles observed in EFCs. These observations suggest that under batch conditions, where flagellum-mediated swimming motility substantially increases the likelihood of cell–electrode contact, the repression of flagellar biosynthesis may limit the initial rate of electrode colonization and consequently delay current generation. Conversely, in EFCs, continuous medium flow likely facilitates physical cell contact with the electrode surface, thereby reducing the dependence on flagellum-mediated motility for initial electrode colonization. Under these hydrodynamic conditions, the *dgcS*-OE strain may develop biofilms and increase current generation more rapidly than the VC strain, presumably because the elevated intracellular c-di-GMP levels promote a transition toward a biofilm lifestyle prior to electrode attachment. However, in addition to medium flow, several environmental factors, including working electrode materials (graphite plate vs. graphite felt) and electrochemical cell configurations, differed between the EFC and batch EC systems used in this study. Moreover, although increased intracellular c-di-GMP levels are known to reduce motility in *S. oneidensis* MR-1 (Ng et al., 2020), flagellum-dependent motility was not directly evaluated for the *dgcS*-OE strain in this study. Therefore, further investigation is needed to determine how these environmental and biological factors influence the initial current profiles of the *dgcS*-OE strain.

In *P. aeruginosa*, the transcription of flagellar biosynthesis genes is regulated by the σ^54^-dependent transcriptional activator FleQ (Hickman and Harwood, 2008). When intracellular c-di-GMP levels are low, FleQ binds to the promoter region of flagellar biosynthesis genes and activates their transcription (Matsuyama et al., 2016). In contrast, elevated c-di-GMP levels promote c-di-GMP binding to FleQ, thereby inhibiting its transcriptional activation capacity and repressing flagellar biosynthesis gene expression (Matsuyama et al., 2016). In *Shewanella* species, the FleQ homolog FlrA functions as a transcriptional activator for the downstream regulator gene *flrC* and other flagellar biosynthesis genes (Gao et al., 2018; Blagotinsek et al., 2020). Therefore, the repression of *flrC* and *flgL* observed in the *dgcS*-OE strain is likely related to c-di-GMP-mediated inhibition of FlrA activity. Furthermore, our RT-qPCR analysis (Figure 4) revealed that increased *dgcS* expression results in downregulation of *flrA* transcription itself, suggesting that decreased FlrA abundance may further contribute to the transcriptional repression of flagellar biosynthesis genes. However, because no σ^54^-dependent promoter-like sequence has been identified upstream of *flrA* (Shi et al., 2014), autoregulation by FlrA appears unlikely. It is therefore conceivable that unknown molecular mechanisms are involved in the regulatory network governing *flrA* transcription in *S. oneidensis* MR-1.

Our study further demonstrated that elevated *dgcS* expression stimulates polysaccharide production (Figure 2G), suggesting that this response contributes to the formation of biofilms with increased biomass by reinforcing the extracellular matrix. In *P. aeruginosa*, FleQ is known to activate the transcription of extracellular polysaccharide (EPS) biosynthesis genes while repressing that of flagellar biosynthesis genes (Hickman and Harwood, 2008; Matsuyama et al., 2016). However, in MR-1, the c-di-GMP-dependent stimulation of EPS production is known to be regulated primarily at the post-transcriptional level (Mukherjee et al., 2020). In this strain, MxdA, a protein harboring an NVDEF motif analogous to the canonical GGDEF domain, functions as a c-di-GMP receptor and stimulates polysaccharide biosynthesis through interaction with the glycosyltransferase MxdB (Thormann et al., 2006; Rakshe et al., 2011; Gambari et al., 2018). Therefore, the enhanced polysaccharide production observed in the *dgcS*-OE strain is likely mediated, at least in part, through this MxdA-dependent regulatory pathway, although experimental verification of this model warrants future study.

In several Gram-negative bacteria, intracellular c-di-GMP and cAMP signaling pathways interact to coordinately regulate diverse cellular processes. For instance, in *Pseudomonas putida*, the c-di-GMP-responsive transcription factor FleQ regulates the expression of the adenylate cyclase gene *cyaA*, leading to decreased cAMP levels in response to elevated c-di-GMP levels (Xiao et al., 2021). Additionally, a crosstalk mechanism between c-di-GMP and cAMP signaling has been reported in *Shewanella putrefaciens*, where a direct interaction between the cAMP-CRP complex and the c-di-GMP effector protein BpfD regulates biofilm formation (Liu et al., 2022; Sun et al., 2025). Our results also demonstrated that *dgcS* overexpression resulted in a slight decrease in the expression of *cyaC* and *crp* (Table 1). Moreover, under batch EC conditions, a slight reduction in intracellular cAMP levels and *mtrC* expression was observed in the *dgcS*-OE strain (Figures 3F, 4). Although these trends were contrary to our initial hypothesis, they may reflect the presence of a potential interaction between c-di-GMP and cAMP signaling in MR-1. Notably, however, under both EFC and batch EC conditions, *dgcS* overexpression did not affect biomass-normalized current generation (Figures 2F, 3D). These observations indicate that the enhanced current generation observed in the *dgcS*-OE strain is primarily attributable to increased colonization of the electrode surface rather than enhanced EET activity per biomass. Accordingly, the contribution of the potential crosstalk between c-di-GMP and cAMP signaling pathways to current generation appears to be limited under the conditions tested. Why intracellular cAMP levels in the *dgcS*-OE strain were reduced specifically under batch EC conditions, while no significant differences were observed under the other two conditions, remains to be elucidated. Further investigation is needed to understand the dynamics of intracellular c-di-GMP and cAMP levels in response to different environmental conditions.

A previous study showed that an engineered MR-1 strain overexpressing a heterologous DGC gene (*E. coli yedQ*) exhibited increased expression of outer membrane *c*-Cyt genes, including *omcA* and *mtrC*, when grown under aerobic conditions (Ng et al., 2020). In contrast, our findings obtained using cells cultured under anaerobic, current-generating conditions suggest that elevated c-di-GMP levels rather weakly repress cAMP levels and related gene expression (Figures 3, 4 and Table 1). Therefore, oxygen availability and intracellular energy levels may influence how DGC expression alters EET-related gene expression in MR-1, although further research is needed to elucidate the underlying molecular mechanisms.

In conclusion, this study demonstrates that the overexpression of the endogenous DGC gene *dgcS* promotes EBF formation and enhances current generation in *S. oneidensis* MR-1. While a previous study reported that overexpressing a heterologous DGC gene (*E. coli yedH*) in MR-1 increased current generation by approximately 2.8-fold under batch conditions in MFCs (Liu et al., 2015), the present study indicates that elevated DGC activity markedly enhances current generation even under medium-flow conditions. This finding suggests the potential utility of this approach for bioelectrochemical processes, including continuous-flow electricity generation and electro-fermentation systems. We anticipate that the insights presented here will contribute to a deeper understanding of c-di-GMP signaling in EAB and facilitate the development of engineering strategies to improve METs through the optimization of electrochemically active biofilms.

## Data Availability

The raw data supporting the conclusions of this article will be made available by the authors, without undue reservation.
