# Supplementary material for: Overexpression of the endogenous diguanylate cyclase gene dgcS enhances biofilm formation by Shewanella oneidensis MR-1 on electrodes
Source: Front Microbiol. 2026 Oct 14;17:1957821. doi: 10.3389/fmicb.2026.1957821 (PMC13713693; doi:10.3389/fmicb.2026.1957821)
Supplement: Supplementary file 1 [file Data_Sheet_1.pdf]

Overexpression of the endogenous diguanylate cyclase gene *dgcS* enhances biofilm formation by *Shewanella oneidensis* MR-1 on electrodes

Nanae Iwanami[1], Akiho Matsumoto[1], Ryotaro Yamamoto[2], Keisuke Tomita[1], Atsushi Kouzuma[1,2*], and Kazuya Watanabe[1]

[1]School of Life Sciences, Tokyo University of Pharmacy and Life Sciences, Hachioji, Japan

[2]Faculty of Life Sciences, Toyo University, Asaka, Japan

Supplementary Material

1 Supplementary Figures and Tables

1.1 Supplementary Figures

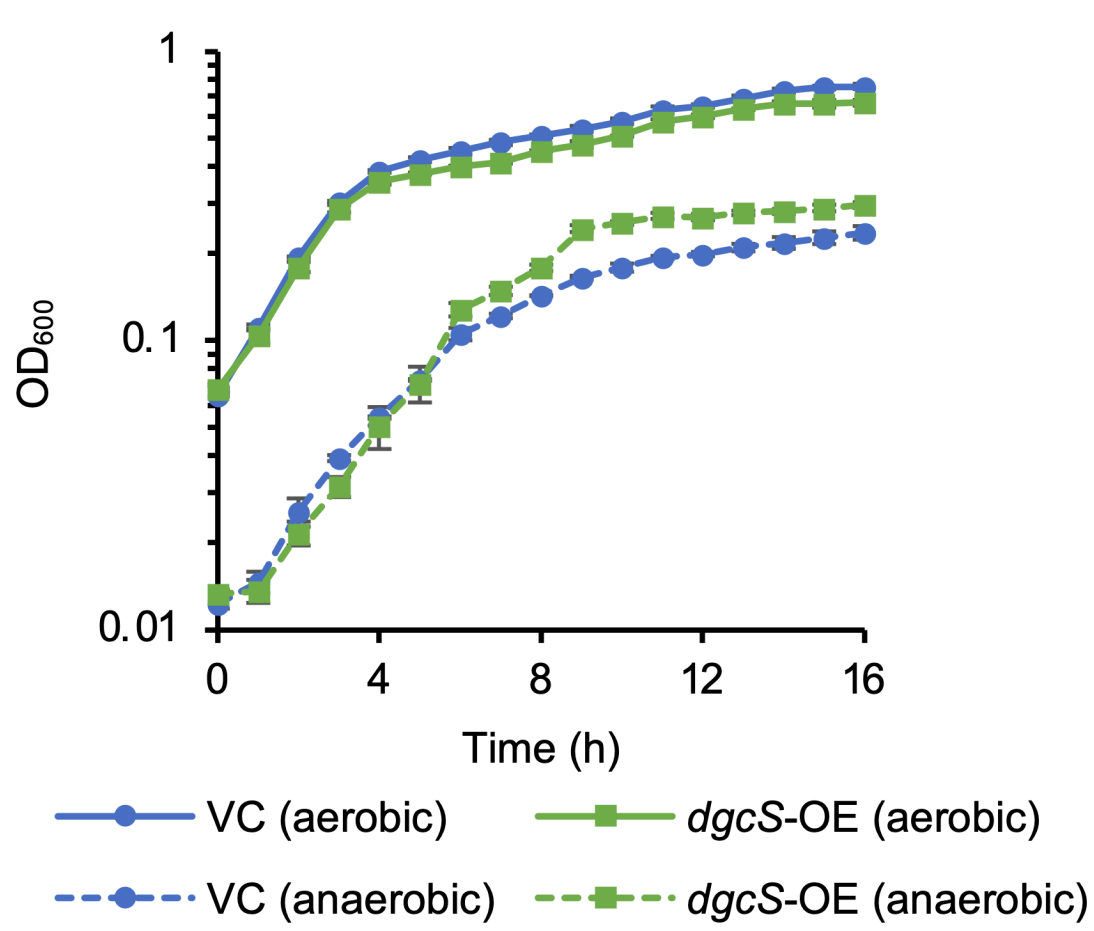

Supplementary Figure 1. Growth of the VC and *dgcS*-OE strains under aerobic and anaerobic fumarate-reducing conditions. Data points and error bars indicate the means and standard deviations, respectively (*n* = 3 biological replicates).

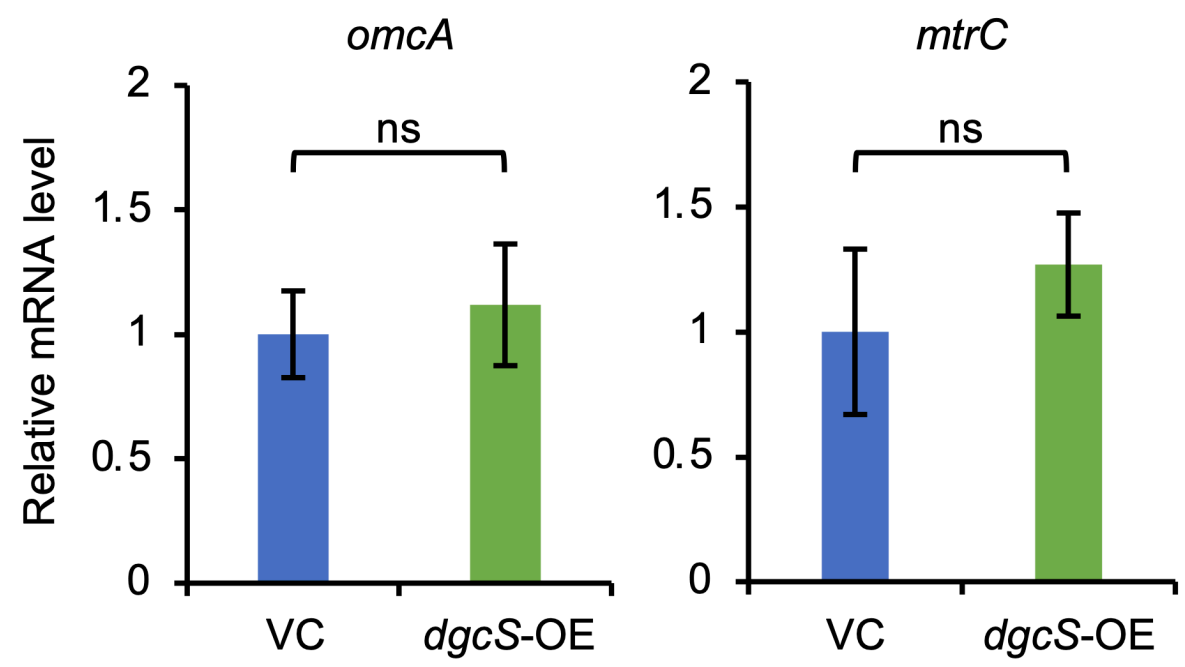

Supplementary Figure 2. RT-qPCR analysis of *omcA* and *mtrC* in the *dgcS*-OE and VC strains grown under fumarate-reducing conditions. Bars and error bars represent the means and standard deviations, respectively (n = 3 biological replicates); ns, not significant ($p > 0.05$; Welch's t-test).

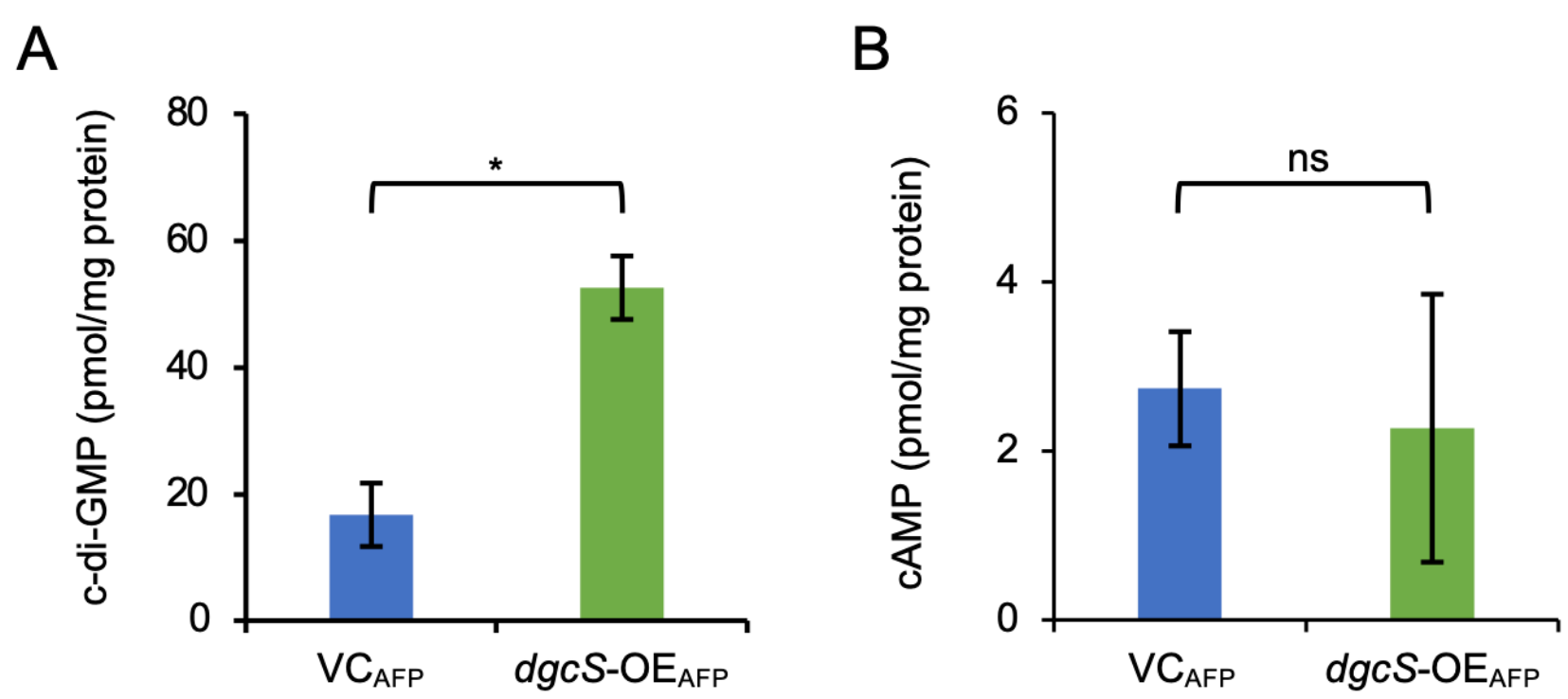

Supplementary Figure 3. Intracellular c-di-GMP level (A) and cAMP level (B) in electrode-associated *dgcS*-OE_{AFP} and VC_{AFP} biofilms. Cells were grown in EFCs for 72 h. Bars and error bars represent the means and standard deviations, respectively (n = 3 biological replicates). Asterisks indicate statistically significant differences ($p < 0.05$; Welch's t-test); ns, not significant.

1.2 Supplementary Tables

Supplementary Table S1. Bacterial strains used in this study.

Strain or plasmid	Relevant characteristic	Source or reference
**Bacterial strains**		
Escherichia coli		
WM6026	Donor strain for conjugation; *lacI*^{q}, *rrnB3*, DE*lacZ4787*, *hsdR514*, DE(*araBAD*)*567*, *E*(*rhaBAD*)*568*, *rph-1*, *att-lambda*::*pAE12*-del(*oriR6K-cat*::*frt5*), DE(*endA*)::*frt*, *uidA*(*delMluI*)::*pir*(wt), *attHK*::pJK1006-*del1/2* (del*oriR6K-cat*::*frt5,* del*trfA*::*frt*)	William Metcalf, University of Illinois
DH5α	F^-, Φ80d*lacZ*ΔM15, Δ(*lacZYA-argF*)U169, *deoR*, recA*1*, *endA*1, *hsdR*17(r_K^-, m_K^+), *phoA*, *supE*44, λ^-, *thi*-1, *gyrA*96, *relA*1	Takara
Shewanella oneidensis		
MR-1	Wild type	ATCC
VC	MR-1 harboring pBBR1MCS-2, Km^r	This study
dgcS-OE	MR-1 harboring pBBR-*dgcS*, Km^r	This study
VC$_{AFP}$	MR-1 harboring pBBR1MCS-2 and pME*lacZ*-AFP, Km^r Gm^r	1
dgcS-OE$_{AFP}$	MR-1 harboring pBBR-*dgcS* and pME*lacZ*-AFP, Km^r Gm^r	This study
cyaC-OE	MR-1 harboring pBBR-*cyaC*, Km^r	2
**Plasmids**		
pME*lacZ*-AFP	Broad-host-range plasmid expressing AFP, Gm^r	3
pBBR1MCS-2	Broad-host-range vector, *lacZ* promoter, Km^r	4
pBBR-*dgcS*	pBBR1MCS-2-based plasmid expressing *dgcS*, Km^r	1
pBBR-*cyaC*	pBBR1MCS-2-based plasmid expressing *cyaC*, Km^r	2

1. Matsumoto A. *et al*. (2021) Identification of a diguanylate cyclase that facilitates biofilm formation on electrodes by *Shewanella oneidensis* MR-1. *Appl. Environ. Microbiol.* 87, e00201-21. doi: 10.1128/AEM.00201-21.
2. Kasai T. *et al*. (2019). Overexpression of the adenylate cyclase gene *cyaC* facilitates current generation by *Shewanella oneidensis* in bioelectrochemical systems. *Bioelectrochemistry* 129, 100-105. doi: 10.1016/j.bioelechem.2019.05.010.
3. Kitayama M. *et al*. (2017) Structures, compositions, and activities of live *Shewanella* biofilms formed on graphite electrodes in electrochemical flow cells. *Appl. Environ. Microbiol.* 83, e00903-17. doi: 10.1128/AEM.00903-17.
4. Kovach M. E. *et al.* (1995) Four new derivatives of the broad-host-range cloning vector pBBR1MCS, carrying different antibiotic-resistance cassettes. *Gene* 166, 175–176. doi: 10.1016/0378-1119(95)00584-1.

Supplementary Table S2. RT-qPCR primers used in this study.

Primer	Sequence (5′–3′)	Target gene
qPCR-dgcS-F	TGCAAGCGATGTTTGATGCG	*dgcS* (SO_1646)
qPCR-dgcS-R	ACGCTGCCCTGTTTTCTTTG	*dgcS* (SO_1646)
qPCR-omcA-F	GGATACGGCGTTGAAGATGT	*omcA* (SO_1779)
qPCR-omcA-R	TGGTATCCGTTCCATTCCAT	*omcA* (SO_1779)
qPCR-mtrC-F	CGGCAATGATGGTAGTGATG	*mtrC* (SO_1778)
qPCR-mtrC-R	GCTTCGTTAGTGGCGAAAAC	*mtrC* (SO_1778)
qPCR-flgL-F	CCAGAAGTGTCGCAGGGAAT	*flgL* (SO_3239)
qPCR-flgL-R	CTGCGTTCAATGCAAGCTGT	*flgL* (SO_3239)
qPCR-flrA-F	GCGGGTGGGTGGTACTAAAA	*flrA* (SO_3232)
qPCR-flrA-R	GGGAACACATTGAGGCGGTA	*flrA* (SO_3232)
qPCR-flrC-F	CTTGCCGCCGTTTACTTAGC	*flrC* (SO_3230)
qPCR-flrC-R	TCGGCGTCATCGGATGTTAG	*flrC* (SO_3230)
qPCR-cyaA-F	CGGCGACTATCATTCTGCT	*cyaA* (SO_4312)
qPCR-cyaA-R	AGCGAGTATCCCCACTCAC	*cyaA* (SO_4312)
qPCR-cyaB-F	ACGGTTGCATCTCACGAA	*cyaB* (SO_3778)
qPCR-cyaB-R	CTTTTTGGGCACGCCTAA	*cyaB* (SO_3778)
qPCR-cyaC-F	TTGGGCCACGTATTTTGCTC	*cyaC* (SO_1329)
qPCR-cyaC-R	TGGATCAAGCGGCTAAAACG	*cyaC* (SO_1329)
qPCR-crp-F	GGGTTCGTGCTAAACAGGCA	*crp* (SO_0624)
qPCR-crp-R	AATGTTTGCGCAATCCGACC	*crp* (SO_0624)
qPCR-16S-F	AGCGCAACCCCTATCCTTAT	16S rRNA
qPCR-16S-R	CGTAAGGGCCATGATGACTT	16S rRNA
